# The Phenomenology of Habits: Integrating First-Person and Neuropsychological Studies of Memory

**DOI:** 10.3389/fpsyg.2018.01176

**Published:** 2018-07-10

**Authors:** Christian Tewes

**Affiliations:** Section of Phenomenology, University Hospital Heidelberg, University of Heidelberg, Heidelberg, Germany

**Keywords:** habits, procedural memory, awareness, goal-directedness, first-person perspective, front-loaded phenomenology

## Abstract

There is an ongoing debate how one can integrate the subjective (first-person) dimension of experiences more thoroughly into neuropsychological research. In cognitive experimental memory research, for instance, cognitive psychology begins by separating the act of recollection from the context where recollections occur, so as to make memory research suitable for study in the experimental conditions of the laboratory. It is the claim of this article that the challenge for memory research consists not merely in the (possible) loss of meaning entailed by transforming embedded recollected experiences into operationalized cognitive functions. Rather, from the outset, the first-person experiential basis of the entire research procedure is often insufficiently elaborated and hence risks neglecting or misrepresenting significant dimensions of the phenomena it studies. I demonstrate this with regard to *habits* understood as *procedural memories*. Research based on the paradigm of embodied cognition and phenomenology has shown that procedural memory-based skills and habits are not necessarily confined to sub-personal (unconscious) processing mechanisms. This paradigm states that some cognitive processes involve not only the brain but also the pre-reflectively experienced lived-body. The key idea is that we have experiential access to bodily processes that are not yet conceptualized or reflexively mediated. In the final part of my paper, I delineate how such experiences can be integrated into the neuropsychological study of habits via the method of ‘front-loaded phenomenology.’

## Introduction

It is the aim of this contribution to show how phenomenological findings about habit formation can be integrated with neuropsychological research. To this end I start by delineating the dominant approach to studying habits in cognitive science, before introducing insights from phenomenology that can helpfully broaden the scope of habits research. How this could be achieved is suggested in the final part of the paper where the method of ‘front-loaded phenomenology’ is explained. This method is especially promising for linking phenomenal experiences with neuropsychological concepts and experiments in a mutually explanatory way.

When I talk of ‘habits’ in this perspective paper, I am mainly referring to ‘procedural skills’ used in concrete action situations, such as the ability to ride a bike or drive a car. Of course, habits comprise a wider range of phenomena than this. Repeated gestures, styles of movements, character traits or addictive behavior are also central facets of habitualized behavior.^1^

Given this, it might surprise a reader that a paper takes as its theme the *experiential* dimension of habits, especially those involved in procedural memory; the term ‘procedural memory’ is traditionally defined in cognitive psychology without reference to *conscious* representation of the previous experiences that resulted in these procedural skills (Schacter, 1987).

This description conforms closely to current research in neuroscience on those habits which arise from processes of procedural learning (skills). Graybiel, for instance, defines habits such as rituals and customs as *originating* in experiene-dependent plasticity. If certain forms of behavior are repeated over a period of days or years, for example the different action sequences required for driving a car, then, according to her, they become fixed and “are performed almost automatically, virtually non-consciously” (Graybiel, 2008, p. 362).

Such definitions of habits are based on empirical research. Priming experiments on word-stem completion or the study of amnesic patients demonstrate that humans are able to acquire habits by means of discriminative learning strategies over a longer period without acquiring any declarative knowledge of these procedural skills. The prevailing view is that these results show that amnesic patients learn discrimination tasks in a way similar to monkeys, namely by gradually reinforcing associations between stimuli and responses (trial and error learning), associations “that develop outside awareness and are rigidly organized” (Bayley et al., 2005, p. 550).

Such descriptions indicate that a major source of research on habit formation is the study of animal behavior. As Dickinson has famously elaborated, two models of behavior dominate existing research on animals. According to the *teleological* model, actions are controlled in the light of the relevant motivational states and consequences of the agent’s activity. This capacity allows the agent a *fluid adaptivity* to changing affordances of the environment. According to the *mechanistic stimulus-response model*, by contrast, an event triggers certain types of activity that have been strengthened by prior experiences (repetitions) and which move an animal to fulfill a certain task without any *explicit knowledge* of the goal thereby achieved (Dickinson, 1985, p. 67–68). This second model is crucial for the prevalent conception of habits in the cognitive sciences (Barandiaran and Di Paolo, 2014, p. 5). In cognitive science experiments, habits are usually understood as the result of activities where the animal loses insight into the relation (the contingencies) between its behavior and its goal (Dickinson, 1985, p. 76). Habits are thus conceived as rigidly performed behavioral patterns that are no longer connected to the intentional achievement of goals. Nevertheless, rigidly performed habits might be beneficial to environmental requirements after all.

These experimental findings and theoretical assumptions go on to form the basis for investigating neural circuits or brain systems in the realization of habits: the transition from-goal directed behavior to habits involve, for instance, a shift in the functional activation of cortico-basal ganglia circuits, a shift of activity of ventral striatal regions to dorsal striatal regions once a learned behavior becomes fixed (Graybiel, 2008, p. 363, 367).

However, as Bernacer and Murillo have recently shown in an Aristotelian-inspired study of habits, this is a specific bias built into this overarching framework of neuropsychological studies on habit formation. It results in a marked tendency to extrapolate – in almost behavioristic fashion – from the results of animal research on procedural memory to humans (Bernacer and Murillo, 2014).

This is not in itself problematic and does not necessarily limit the research paradigm. The question is whether this approach actually sheds light on *every major aspect* of habit formation. For an analysis and investigation of automatically triggered stereotyped behavior, this approach certainly delivers fruitful insights into their processing mechanisms. Further investigation into these mechanisms and their neural underpinnings might also help explain obsessive-compulsive disorders such as Tourette syndrome or addictive behavior which are beyond intentional control (Graybiel, 2008, p. 374).

However, it is important to bear in mind that in the case of human habits, further cognitive resources might influence the realization of procedural memories, such that limiting oneself to the findings of animal research could neglect further significant aspects of *culturally embodied habits*.

Let us summarize the main argument so far: if the standard definition of habits takes them to be rigid, insensitive to the outcome of actions (not goal-directed) and indeed unconscious, then habits become divorced from the experiential dimensions of the first-person perspective and from intentional actions. There are good reasons to think that such a definition is one-sided. As I will explain in the next section, phenomenological- and embodiment-inspired research on habits provide a very useful antidote here, bringing to light that awareness and intentionality play a vital role in the constitution of human habits.

## Broadening the Scope of Habits: Awareness, Adaptivity, and Goal-Directedness

One way to broaden the explanatory scope of habits in the cognitive sciences is to study in more detail how the personal level of experience contributes to the manifestation of habits, just as it contributes to the constitution of perceptual content (Noë, 2012, p. 32–33). This requires focusing on the experiential aspects of habitualized processes, a procedure that is excluded from the outset by the prevailing definition of habit formation. Research on skill-based behavior has already shown that the acquisition and performance of implicit knowledge cannot be reduced to automatically triggered stereotyped behavior. As Sun et al. have convincingly shown over a decade ago, complex skills often require important interactions between implicit and explicit knowledge (Sun et al., 2005).

Phenomenological studies have proved especially useful in highlighting aspects hitherto neglected in the study of habits. Drawing upon the phenomenological research tradition, for instance, Edward Casey has defined habits as “[a]n active immanence of the past in the body that informs present bodily actions in an efficacious, orienting, and regular manner” (Casey, 2000, p. 149). Casey’s key insight is that for these properties to emerge, there needs to be a kind of pre-reflexive awareness and sometimes even *intensified attention* on the part of the subject, such that the resulting actions are adaptive and situationally adequate (goal-directed).

A familiar everyday example can illustrate this. An experienced car driver is conversing animatedly with her passenger during a journey. At the same time she is smoothly regulating her speed, direction of travel and the distance between her car and other vehicles. She does not need to represent her ongoing actions in a reflexive manner (steering the wheel, shifting gears, observing the surrounding traffic by means of rear-view mirrors) but rather she *re-*enacts them in the present. In this context, ‘re-enacting’ means that directly perceived affordances of an action situation determine to a great extent the resulting sensorimotor interactions. The latter are not specified beforehand in an explicit conceptual format (Gallagher, 2008, p. 536).

Very often, the perceived motor goals of her action are the product of earlier interactions and learning processes that result in *efficacious* habits, such as avoiding a collision with oncoming vehicles. In many case, this successful application (situation-dependent manifestation) of habits requires – as is obvious in complex traffic situations – at least a minimal and sometimes intensified form of awareness. This already points to the significant role that contextually embedded habits play in the cognitive architecture of the human mind and one that cannot be equated with stereotyped or rigidly automatic behavior.

That this is the case is explained by Sutton et al. (2011) in a paper on why habits and skills are more than reflexes devoid of flexible goal-directed behavior. Sutton et al. (2011) general claim is that a kind of mindedness in habitual acts can exist which is neither the outcome of reflexive behavior nor of purely sub-personal mechanisms. To take an example, many athletes such as table tennis players or elite cricketers, need to react within fractions of a second in order to hit the ball at a specific angle, and they do so depending on the “experience of playing *this* fast bowler in *these* conditions, and on dynamically updated awareness of the current state of the match and of the opposition’s deployment” (Sutton et al., 2011, p. 80). Drawing on these and similar examples, Sutton et al. (2011, p. 80) conclude that the movement of the cricketer or table-tennis player is “fast enough to be a reflex, yet it is *perfectly* context sensitive.”. This fits neatly with what Casey called the ‘orienting role’ of habits. It is possible for the expert (a) to select between different skills and habits in accordance with the requirement of the concrete situation and (b) to *adapt* the action scheme they finally choose (for example, hitting the ball with top-spin) to the entire action scenario as it unfolds. The constant adaptation to unfolding scenarios by professionals in sports or other areas of expertise is highly attention-based yet does not interrupt their skillful coping with the environment. It implies that agents very often do have an experience-based access to the manifestation of their habits, enabling them to *modify* their activities *at will* in the light of perceived affordances (Mann et al., 2007, p. 458). This is a decisive difference between *adaptively* performed and *stereotyped* habits. The former are integrated into a modifiable realization of an intentional achieved goal. Assuming that these phenomenological and conceptual extensions of habits are correct, how should we integrate these phenomenological insights of habit formation into research in the cognitive sciences?

## Integrating Phenomenology Into Neuropsychological Research

The characteristics of habit formation delineated above are not to be dismissed as folk-psychological descriptions. Phenomenology in the Husserlian sense aims at discovering essential structures of phenomenal experiences. One can differentiate here between the pre-reflexive conscious awareness of “living through” everyday experiences and the *specification* of these experiences from a reflexive stance, the so-called ‘phenomenological reduction’ (Vermersch, 2009). After having suspended the natural attitude toward everyday experiences, the next step in a phenomenological analysis is to find, in a quasi-mathematical spirit, the *ideal possibilities* or conceptual structures involved in these experiences (Marbach, 2007, p. 388). These ideal possibilities are identified by considering each of the invariant properties and conditions of these experiences (Fuchs, 2016, p. 5), a procedure Casey undertakes in the case of habits. This methodological procedure is accomplished from the first-person perspective of experience (Petitmengin and Bitbol, 2009) and it is important to highlight that the concrete findings of such a procedure are open to falsification.

It is crucial for a phenomenological analysis that its outcomes are *accessible*, *understandable*, and *evaluable* from the first- and the second-person perspective as well (Bitbol and Petitmengin, 2013, p. 277). It is possible, on the one hand, to communicate insights obtained from the first-person perspective by means of the description and conceptualizing of the underlying experiences. On the other hand, the conceptual content of those descriptions can function as a tool for others to *redirect* their own focus of attention to the specified phenomena. This procedure enables other researchers to evaluate the results presented. This intertwinement of first- and second-person perspective opens up possibilities for connecting phenomenological investigations with the research methods and findings of the cognitive sciences that take the third-person perspective, for instance neurological research.

There are other methods which can be used to obtain reliable results from the first-person perspective of experience. One such method, particularly useful for investigating the skills which an expert displays, is the ‘thinking-aloud protocol technique.’ In this method participants voice their thoughts as they complete a cognitive task, such as making calculations or playing chess. Combined with the ‘expert-performance method,’ where experts are asked to perform specific challenges, it is possible to analyze the structure of expertise in specific domains (Ericsson, 2006, p. 231).

Such methods are not purely behavioral but require that the subject has at least a minimal experiential access to the thought processes they are simultaneously reporting (Gallagher, 2010, p. 22–23). How exactly the thinking-aloud protocol technique, introspection and phenomenological methods relate to each other is presently a major research issue, one that goes beyond the scope of this paper. Nevertheless, it is reasonable to assume that these methods and their findings are not mutually exclusive, each having their own strengths and limitations and each capable of being integrated with the study of consciousness, just as their various claims (including those of phenomenology) must be revisable on the basis of further evidence (Yoshimi, 2016, p. 300).

How is it possible to integrate the first- and third-person methods and their findings in studying consciousness? One principle challenge here is to show how first-person experience, such as consciously realized habits and third-person data, can be related in an explanatory way to the neural and behavioral level without losing the psychological phenomenon in question (Kotchoubey et al., 2016, p. 8). One solution, I suggest, is to be found in Shaun Gallagher’s concept of ‘front-loaded phenomenology,’ an approach which bridges the gap between different experiential and conceptual perspectives and the corresponding research methods. The idea behind Gallagher’s approach is to use phenomenologically gained insights to frame and guide experimental designs in neuropsychology (Gallagher, 2003, p. 91).

Front-loaded phenomenology has a complement in experimental neurophenomenology, where subjects practice first-person methods in order to heighten their attention toward mental phenomena. Based on newly disclosed experiences and their conceptualization, the aim is to uncover the corresponding physiological data in neurophysiological studies (Thompson, 2007, p. 339). Front-loaded phenomenology, by contrast, uses such existing phenomenological findings to *inform the setup* of psychophysical experiments. To give an example, one can differentiate, from a phenomenological perspective, between (a) movements that occur to me when somebody raises my arm, and (b) self-initiated movements such as reaching for a cup. In Gallagher’s terms, we can distinguish a *sense of ownership* (the movement is occurring to me) and a *sense of agency* (Gallagher, 2003, p. 92). This distinction has been used to set up psychophysical experiments that uncovered distinct neurological activities in each case: movements attributable to an initiation *by* the self are associated with the anterior insula bilaterally whereas movements that merely happen *to* me are associated with activities in the right inferior parietal lobe (Farrer and Frith, 2002, p. 601).

When applied to our topic, these more finely grained characteristics of habit formation suggest that their manifestation can also involve goal-directed and consciously performed activities (Bernacer et al., 2014). Analogous to the distinction between the sense of ownership and the sense of agency, one can expect attention-based adaptively performed habits to involve different neural networks than those involved in stereotyped habits.

In this light, a hypothesis can be formulated: that the neural network which is related to attentional and executive control is probably involved not only in learning new skills but also in the performance of consciously realized habits. There already exists extensive research in neuroscience on how experience-based practice changes the human brain (Kelly and Garavan, 2005) and how skill acquisition is related to different learning phases at the cognitive and neural level (Tenison et al., 2016). Empirical studies, however, have yet to investigate the realization of habitual activities in action situations which depend on *intensified attentional capacities*. Psychophysical experiments might therefore be designed to study adaptive habits in terms of the interrelationship between the cortical areas involved in executive functions (Rueda et al., 2005, p. 578) and the cortico-basal ganglia circuits that are associated with the automatic execution of movement and motor learning (DeLong and Wichmann, 2010). In more concrete terms, one could devise an experiment to compare the neural activities of subjects performing a habitualized activity, such as walking in a manner attentive to their own kinesthetic patterns, and subjects who are simultaneously walking and daydreaming. A recent pilot study using a prototype wearable PET (positron emission tomography) helmet has demonstrated that even deep brain structures such as the basal ganglia circuits are, at least in principle, examinable during a variety of movement tasks (Bauer et al., 2016), indicating that the sort of experiment suggested here may soon (if not already) be feasible.

## Conclusion

The results of such phenomenological-informed neuropsychological research projects would, of course, be open to falsification. We can front-load the phenomenology of habits and frame the hypothesis that (attention-based) adaptively performed habits will involve different brain activity than stereotyped habits. That might turn out to be wrong. However, this does not mean that neuroscience has the sole authority to decide the validity of phenomenological outcomes. Phenomenology reaches findings by means of controlled methods from the first- and second-person perspective that have their own scientific justification (Bitbol and Petitmengin, 2013, p. 277). The distinction between stereotyped and attention-based (adaptive) habits in the scientific literature is highly plausible and its existence is not in need of a confirmation at the neural level of description. Nevertheless, phenomenological findings are clearly open to critical evaluation and correction by other disciplines, including cognitive psychology. In this sense, phenomenological insights and methods can be part of a general process of *theory building*, as we have seen here in the case of habit formation, one whose aim is to integrate goal-directed behavior and automatically induced habits (Dezfouli and Balleine, 2013). By combining phenomenological, behavioral, and neuroscientific methods, we can begin to develop a richer and more coherent theory of habits than has previously been possible, one which studies the distinction between adaptive and stereotyped habits in terms of the way they are experienced, their behavioral expressions, and their neural underpinnings.

## Author Contributions

The author confirms being the sole contributor of this work and approved it for publication.

## Conflict of Interest Statement

The author declares that the research was conducted in the absence of any commercial or financial relationships that could be construed as a potential conflict of interest. The reviewer AHD declared a shared affiliation, with no collaboration, with the author to the handling Editor.

## Footnotes

1 Thus in the phenomenological tradition, Husserl, for instance, uses the concept of ‘Habitus’ to refer to human comportment or manner, characteristics formed by sedimented (routinized) actions, beliefs and personal styles (Moran, 2011, p. 59).

## References

[B1] Barandiaran X. E. Di Paolo E. A. (2014). A genealogical map of the concept of *habit*. *Front. Hum. Neurosci.* 8:522. 10.3389/fnhum.2014.00522 25100971 PMC4104486

[B2] Bauer C. E. Brefczynski-Lewis J. Marano G. Mandich M.-B. Stolin A. Martone P. (2016). Concept of an upright wearable positron emission tomography imager in humans. *Brain Behav.* 6:e00530. 10.1002/brb3.530 27688946 PMC5036439

[B3] Bayley P. J. Frascino J. C. Squire L. R. (2005). Robust habit learning in the absence of awareness and independent of the medial temporal lobe. *Nature* 436 550–553. 10.1038/nature03857 16049487 PMC1457096

[B4] Bernacer J. Balderas G. Martinez-Valbuena I. Pastor M. A. Murillo J. I. (2014). The problem of consciousness in habitual decision making. *Behav. Brain Sci.* 37 21–22. 10.1017/s0140525x13000642 24461349

[B5] Bernacer J. Murillo J. I. (2014). The Aristotelian conception of habit and its contribution to human neuroscience. *Front. Hum. Neurosci.* 8:883. 10.3389/fnhum.2014.00883 25404908 PMC4217385

[B6] Bitbol M. Petitmengin C. (2013). A defense of introspection from within. *Constr. Found.* 8 269–279.

[B7] Casey E. (2000). *Remembering. A Phenomenological Study*, Bloomington, IN: University Press.

[B8] DeLong M. Wichmann T. (2010). Changing views of basal ganglia circuits and Circuit disorders. *Clin. EEG Neurosci.* 41 61–67. 10.1177/155005941004100204 20521487 PMC4305332

[B9] Dezfouli A. Balleine B. W. (2013). Actions, action sequences and habits: evidence that goal-directed and habitual action control are hierarchically organized. *PLoS Comput. Biol.* 9:e1003364. 10.1371/journal.pcbi.1003364 24339762 PMC3854489

[B10] Dickinson A. (1985). Actions and habits: the development of behavioural autonomy. *Philos. Trans. R. Soc. B Biol. Sci.* 308 67–78. 10.1098/rstb.1985.0010

[B11] Ericsson K. A. (2006). “Protocol analysis and expert thought: concurrent verbalizations of thinking during experts performance on representative tasks,” in *The Cambridge Handbook of Expertise and Expert Performance*, eds Ericsson K. A. Charness N. Feltovich P. Hoffman R. R. (Cambridge: Cambridge University Press), 223–242.

[B12] Farrer C. Frith C. (2002). Experiencing oneself vs another person as being the cause of an action: the neural correlates of the experience of agency. *Neuroimage* 15 596–603. 10.1006/nimg.2001.1009 11848702

[B13] Fuchs T. (2016). “Anthropologische und phänomenologische Aspekte psychischer Erkrankungen,” in *Psychiatrie, Psychosomatik Psychotherapie*, eds Möller H.-J. Laux G. Kampfhammer H.-P. (Berlin: Springer), 1–15.

[B14] Gallagher S. (2003). Phenomenology and experimental design. *J. Conscious. Stud.* 10 85–99.

[B15] Gallagher S. (2008). Direct perception in the intersubjective context. *Conscious. Cogn.* 17 535–543. 10.1016/j.concog.2008.03.003 18442924

[B16] Gallagher S. (2010). “Phenomenology and non-reductionist cognitive science,” in *Handbook of Phenomenology and Cognitive Science*, eds Gallagher S. Schmicking D. (Dordrecht: Springer), 21–34. 10.1007/978-90-481-2646-0_;2

[B17] Graybiel A. M. (2008). Habits, rituals and the evaluative brain. *Annu. Rev. Neurosci.* 31 359–387. 10.1146/annurev.neuro.29.051605.112851 18558860

[B18] Kelly A. C. Garavan H. (2005). Human functional neuroimaging of brain changes associated with practice. *Cereb. Cortex* 15 1089–1102. 10.1093/cercor/bhi005 15616134

[B19] Kotchoubey B. Tretter F. Braun H. A. Buchheim T. Draguhn A. Fuchs T. (2016). Methodological problems on the way to integrative human neuroscience. *Front. Integr. Neurosci.* 10:41 10.3389/fnint.2016.00041 PMC5126073 27965548

[B20] Mann D. T. Williams A. M. Ward P. Janelle C. M. (2007). Perceptual-cognitive expertise in sport: a meta-analysis. *J. Sport Exerc. Psychol.* 29 457–478. 10.1123/jsep.29.4.457 17968048

[B21] Marbach E. (2007). Towards integrating husserlian phenomenology with cognitive neuroscience of consciousness. *Synth. Philos.* 44 385–400.

[B22] Moran D. (2011). Edmund Husserl’s phenomenology of habituality and habitus. *J. Br. Soc. Phenomenol.* 42 53–77. 10.1080/00071773.2011.11006731

[B23] Noë A. (2012). *Varieties of Presence.* Cambridge, MA: Harvard University Press 10.4159/harvard.9780674063013

[B24] Petitmengin C. Bitbol M. (2009). The validity of first-person descriptions as authenticity and coherence. *J. Conscious. Stud.* 16 363–340.

[B25] Rueda M. Posner R. Rothbart M. K. (2005). The Development of Executive Attention: contributions to the emergence of self-regulation. *Dev. Neuropsychol.* 28 573–594. 10.1207/s15326942dn2802_;2 16144428

[B26] Schacter D. (1987). Implicit memory: history and current status. *J. Exp. Psychol.* 13 501–518. 10.1037/0278-7393.13.3.501 8689472

[B27] Sun R. Slusarz P. Terry C. (2005). The interaction of the explicit and the implicit in skill learning: a dual-process approach. *Psychol. Rev.* 112 159–192. 10.1037/0033-295X.112.1.159 15631592

[B28] Sutton J. Mcilwain D. Christensen W. Geeves A. (2011). Applying intelligence to the Reflexes: embodied skills and habits between Dreyfus and Descartes. *J. Br. Soc. Phenomenol.* 42 78–102. 10.1080/00071773.2011.11006732

[B29] Tenison C. Fincham J. M. Anderson J. R. (2016). Phases of learning: how skill acquisition impacts cognitive processing. *Cognit. Psychol.* 87 1–28. 10.1016/j.cogpsych.2016.03.001 27018936

[B30] Thompson E. (2007). *Mind in Life. Biology, Phenomenology and the Sciences of Mind.* Cambridge, MA: Harvard University Press.

[B31] Vermersch P. (2009). Describing the practice of introspection. *J. Conscious. Stud.* 16 20–57. 16650595

[B32] Yoshimi J. (2016). “Prospects for a naturalized phenomenology,” in *Philosophy of Mind and Phenomenology*, eds Dahlstrom D. Élpidorou A. Hopp W. (New York, NY: Routledge).

